# The Effect of Thoracic Radiotherapy on the Quality of Life in Lung Cancer Patients

**DOI:** 10.7759/cureus.13870

**Published:** 2021-03-13

**Authors:** Ayfer Ay Eren, Mehmet F Eren, Sedat Koca

**Affiliations:** 1 Radiation Oncology, Radiation Oncology Clinic, Saglik Bilimleri University, Kartal Dr. Lutfi Kirdar Education and Research Hospital, Istanbul, TUR; 2 Radiation Oncology, Marmara University Pendik Education and Research Hospital, Istanbul, TUR; 3 Radiation Oncology Clinic, Bahçeşehir University Medical Park Göztepe Hospital, Istanbul, TUR

**Keywords:** radiotherapy, quality of life, lung cancer

## Abstract

Introduction

This study aimed to investigate changes in respiratory symptoms and quality of life (QoL) in patients with locally advanced and metastatic lung cancer receiving thoracic radiotherapy (RT). We investigated the correlation between the level of symptom relief and tumor response.

Methods

Thirty-two patients were included in this study. The European Organization for Research and Treatment of Cancer (EORTC) Quality of Life (QoL) Questionnaire (QLQ)-C30 and EORTC QLQ-LC13 were used to investigate QoL changes. Assessments were performed on the first day of RT, on the last day of RT, routinely monthly follow-ups, and three months after RT.

Results

The median age of the patients was 62; 88% of the patients were male. For the symptom scale, fatigue and dyspnea provided significant improvement at the end of RT (p=0.000, p=0.047). No significant improvement was observed at the end of RT in pain and insomnia. While coughing showed substantial improvement at the end of RT (p=0.004), the maximum improvement was achieved during the third-month follow-up (p<0.001). No significant improvement was observed at the end of RT in hemoptysis, but a considerable improvement was observed during the third-month follow-up (p=0.008).

Conclusion

This study confirms that RT offered palliation of respiratory symptoms and improved QoL in a substantial proportion of patients with lung cancer.

## Introduction

Lung cancer is the most commonly diagnosed malignancy globally. It is also responsible for 19.4% of all cancer deaths [1]. This makes it the most common cause of cancer-related death in men and the second most common in women after breast cancer. Most cancers that start in the lung, known as primary lung cancers, are carcinomas. The two main types are small-cell lung carcinoma (SCLC) and non-small cell lung carcinoma (NSCLC). The most common symptoms are coughing, weight loss, shortness of breath, and chest pain. The treatment strategies for locally advanced lung cancer usually consist of radiotherapy (RT), with or without chemotherapy, for various purposes such as radical, postoperative, preoperative, or palliative care. RT improves local control (LC), overall survival (OS), and QoL in patients with the local disease [2].

In recent years, technical developments in RT (three-dimensional conformal radiotherapy (3D-CRT), intensity-modulated RT (IMRT), and stereotactic body RT (SBRT)) allow radiotherapy to be administered with less toxicity and in smaller areas. Technical developments are also associated with reducing side effects and improving QoL. QoL is the satisfaction and happiness of individuals in the regions that are important to them. Health-related QoL (HRQoL) covers the versatile joy and happiness of an individual in the living spaces that affect or are affected by their health [3]. Response rate or survival time alone is not a criterion for evaluating the treatments administered. Treatment should aim to improve the QoL and extend the survival time because of the palliation of symptoms.

Here, questioning the QoL associated with health has become an essential criterion for evaluating the palliative effect of treatment or problems related to treatment and deciding on a clinical course. The European Organization for Research and Treatment of Cancer (EORTC) has developed question scales to evaluate the health-related overall QoL of cancer patients (EORTC QLQ-C30) [4]. QoL question scales have been translated into many languages worldwide. Their accuracy and reliability have been proven in many studies [5]. EORTC has also created a questionnaire to evaluate symptoms and QoLs associated with diseases specific to much cancer and QLO-30 related to overall health. The lung cancer symptom scale (LCSS = QLQ-LC13) questionnaire is one of them [6]. This scale aims to evaluate the symptoms affecting the patient. Therefore, this study aims to investigate the overall and specific effect of treatment on QoL in patients who receive thoracic radiotherapy with Karnofski and Eastern Cooperative Oncology Group (ECOG) performance scales [7].

## Materials and methods

Thirty-two patients treated with thoracic RT for curative or palliative purposes were enrolled in this study from January 2009 to June 2009. Ethics committee approval for the study was obtained from Istanbul University Cerrahpaşa Faculty of Medicine (05/01/2009). The criteria to select patients were as follows: patients diagnosed with terminal lung cancer with a pathology report, with a Karnofsky performance score (KPS) above 50%, with an expectation of survival of at least eight weeks. It was observed that three of the patients died during the treatment, one died at the end of the treatment, and one patient left the treatment. During the study, patients were assessed using the QLQ-C30 and QLQ-LC13 QoL questionnaires, KPS, ECOG scale, and Radiation Therapy Oncology Group (RTOG) side-effects scale. Patients filled the questionnaire forms and scales on the first day of RT, on the last day of RT, during the first three months after RT, and routinely monthly follow-ups. Patients were also evaluated using RTOG criteria for acute and late toxicity during weekly follow-ups [8]. According to tumor shrinkage, patients' tumor response after RT was objectively assessed using Response Evaluation Criteria in Solid Tumors (RECIST) version 1.1 (v1.1) [9]. Responses according to RECIST v1.1: a) Complete response: Disappearance of all target lesions and pathological lymph nodes, b) Partial response: At least a 30% decrease in the sum of the lesions, c) Stable disease: Neither sufficient shrinkage nor sufficient increase as a progressive disease, d) Progressive disease: At least a 20% increase in the sum of the present lesions, the appearance of new lesions, and at least 5 mm increase.

RT planning of patients was conducted using a linear accelerator device with three-dimensional conformal radiotherapy. Definitions of gross tumor volume (GTV), clinical target volume (CTV), and planned target volume (PTV) were based on International Commission on Radiation Units and Measurements (ICRU) 50 and 62, 45-60 Gy dose as 1.8-2 Gy daily was administered [10]. Our study's primary endpoint was HRQoL, and it was evaluated with two measurements: the EORTC QLQ-C30 questionnaire (version 3.0) with a lung cancer symptom scale EORTC QLQ-LC13. EORTC QLQ-C30 version 3.0 contains 30 cancer-specific questions composed of five functional scales (physical, role, cognitive, emotional, and social), three symptom scales (fatigue, pain, and nausea/vomiting), global health and QoL scales, and several single items assessing the most common symptoms of cancer patients (dyspnea, appetite loss, insomnia, constipation, and diarrhea) and perceived financial impact. The EORTC QLQ-LC13 comprises 13 questions describing specific symptoms: dyspnea, cough, hemoptysis, dry mouth, dysphagia, alopecia, chest pain, pain in the arm or shoulder, and pain in other body parts. All essential function scales and scores of individual items were transformed from 0 to 100. For the functional and global health status scales, higher scores mean a better level of functioning. For the symptom scales, higher scores represent greater symptoms.

Statistical analysis

The nonparametric Mann-Whitney and Wilcoxon signet test is used to test the treatment effect between the groups. Differences between QoL and KPS and ECOG performance were conducted by correlation analysis.

## Results

Thirty-two patients were included in our study. The median age of the patients was 62 (50-80). Eighty-eight percent of the patients were male. While 14 patients received concurrent chemoradiotherapy, six received neoadjuvant chemotherapy. On the first day, on the last day of therapies, and during the first, second, and third-month follow-ups of patients, QLQ-C30 and QLQ-LC13 questionnaires, KPS, and ECOG performance evaluations were carried out for all patients. According to RTOG side-effects grading, acute toxicities during therapies and follow-ups of patients were evaluated. Acute skin toxicity was observed in five patients (GR I in 3 cases, GR II in 1 case, GR III in 1 case). Dysphagia was observed in 71% of the patients, GR I in eight patients, GR II in 17 patients, and GR III in one patient. During the third-month follow-up, it was observed that all acute toxicities healed spontaneously. The tumor response of patients after RT was objectively evaluated according to tumor shrinkage. According to the third-month follow-up results, we observed complete response in 22 patients, partial response in five patients, stable disease in three patients, and progression in two patients. The global health status was statistically significant at the end of RT as compared to the beginning of RT, and it was observed that this situation continued in the third month after RT (p<0.000, p=0.000, respectively). When the functional scale was examined, the physical and role scales increased significantly at the end of RT as compared to the beginning of RT (p=0.005, p=0.002, respectively). For the physical and role scales, the maximum significant value was reached at the end of RT in the third month as compared to the beginning of RT (p=0.000). On the emotional scale, while there was no significant increase at the end of RT as compared to the beginning of RT (p=0.019), substantial improvement was shown in the third month after RT (p=0.001). The cognitive functions were examined; there was no significant change at the beginning and end of RT and in the third month after RT (p=0.010, p=0.025, respectively). For the social scale, a significant improvement was observed at the end of RT as compared to the beginning (p=0.009). In contrast, there was no statistical significance in the third month after RT (p=0.013). When the components of the symptom scale were examined, it was observed that fatigue and dyspnea provided maximum significant improvement at the end of RT as compared to the beginning of RT, and this situation continued in the third month after RT (p=0.000, p=0.047, p=0.053, respectively). While no significant improvement was observed at the end of RT in pain and insomnia (p=0.048, p=0.058, respectively), it was found that substantial progress was achieved in the third month after RT (p=0.001, p=0.007, respectively). No significant improvement was observed in nausea/vomiting, appetite loss, constipation, diarrhea, and financial difficulties associated with RT. QLQ-C30 and QLQ-LC13 scores and median, standard deviation, and p-values at the beginning of the RT, at the end of RT, and in the third month are summarized in Table 1. It was observed that the dyspnea scales of both QLQ-C30 and QLQ-LC13 reached maximum improvement at the end of RT as compared to the beginning of RT, and this improvement continued in the third-month follow-ups as compared to the beginning of the treatment (p=0.000). QLQ-C30 and QLQ-LC13 were found to be correlated in the dyspnea scale. While coughing showed significant improvement at the end of RT (p=0.004), the maximum improvement was achieved during the 3rd-month follow-up (p <0.001). There was no significant improvement observed at the end of RT in hemoptysis (p=0.056), and a considerable improvement was observed during the 3rd-month follow-up (p=0.008). While there was no improvement in dysphagia at the end of RT (p=0.221), it was found that there was a complete improvement in all patients during the third-month follow-ups (p=0.000). In terms of scales of dry mouth, peripheral neuropathy, alopecia, arm pain, and pain in other body parts, no improvement was observed either at the end of RT or during the third-month follow-ups as compared to before RT.

**Table 1:** Median, standard deviation, and p-values at the beginning of the RT, end of the RT, and in the third month for QLQ-C30 and QLQ-LC13 scores RT: radiation therapy; QLQ-C30: quality of life of cancer patients; QLQ-LC13: quality of life questionnaire lung cancer module

	Beginning of the RT			End of the RT			3rd month after RT		
	Median	Std deviation	p	Median	Std deviation	p	Median	Std deviation	p
Global Health Status	50.00-15.74		0.000	83.30-18.34		-	83.30-12.80		0.000
Functional Scale									
Physical	69.95-20.13		0.002	93,30-16.96		-	100.00-16.35		0.000
Role	66.60-20.74		0.005	100.00-16.55		-	100.00-13.40		0.000
Emotional	66.60-25,38		0.019	70.80-23.06		-	100.00-20.74		0.001
Cognitive	83.30-18.33		0.10	100.00-13.25		-	100.00-16.39		0.025
Social	74.80-17.18		0.013	100.00-16.98		-	100.00-19.30		0.013
Symptom scale									
Fatigue	38.65-17.80		0.000	33.30-17.32		-	16.65-22.81		0.000
Nausea/Vomiting	0.00-22.94		0.306	0.00-18.88		-	0.00-14.17		0.047
Pain	33.30-26.67		0.048	24.95-23.35		-	0.00-19.03		0.001
Dyspnea	66.60-33.23		0.000	16.50-25.03		-	0.00-18.79		0.000
Insomnia	0.00-23.93		0.058	0.00-21.47		-	0.00-14.03		0.007
Pain	0.00-33.44		0.322	0.00-25.36		-	0.00-16.92		0.053
Constipation	0.00-20.47		0.644	0.00-21.93		-	0.00-20.61		0.822
Diarrhea	0.00-0.00		1.0	0.00-0.00		-	0.00-0.00		1.0
Financial Difficulties	0.00-29.23		0.306	0.00-25.30		-	0.00-25.35		0.272
QOL-LC13									
Dyspnea	44.40-21.62		0.000	11.10-18.79		0.000	11.10-19.30		0.000
Cough	66.60-33.58		0.004	33.30-26.41		0.001	33.30-21.34		0.000
Hemoptysis	0.00-30.50		0.056	0.00-19.74		0.038	0.00-17.68		0.004
Dry mouth	0.00-26.76		0.088	0.00-17.40			0.00-16.35		0.039
Dysphagia	0.00-21.96		0.000	33.30-29.73		1.00	0.00-16.90		0.078
Neuropathy	0.00-23.71		0.341	0.00-21.13		0.03	0.00-11.19		0.03
Alopecia	0.00-21.96		0.705	0.00-22,39			0.00-22.39		0.739
Chest pain	33,30-33,85		0.007	16.65-20.61		0.004	0.00-18.65		0.000
Pain in arm/shoulder	0.00-29.14		0.017	0.00-14.65		0.019	0.00-14.91		0.003
Pain in the body	0.00-25.36		0.134	0.00-17.40			0.00-18.40		0.059

## Discussion

The EORTC QLQ-C30 is a widely used QoL assessment form worldwide. EORTC QLQ-LC13 is specific to lung cancer, and many studies on QoL in lung cancer have been conducted over the years [6]. According to chemotherapy use and stage, QoL studies, including radiotherapy on lung cancer, were performed separately for NSCLC and SCLC. The performance statuses of patients were evaluated using ECOG and KPS. In our study, similar to other studies, it was shown that there is a significant correlation between QLQ-C30 and QLQ-LC13 in patients with lung cancer to whom curative RT is administered [11-13]. A recent study showed that the relationship between KPS and QLQ-C30 was not significant [14]. Guzelant et al. evaluated QLQ-C30 and KPS's correlation in lung cancer patients and showed that KPS was positively correlated with functional scale, role scale, and fatigue in the Turkish population [15]. In our study, similar to the study conducted by Yucel et al. [16], KPS was found to be associated with functional and role scales.

In contrast, KPS was not associated with fatigue. When the tumor responses were evaluated, it was shown in the study of Pujol et al. that QoL was positively associated with tumor response and as the diameter of the tumor shrinks [17]. In 22 patients with complete response according to RECIST v1.1 in the third-month follow-up chest computed tomography (CT), a significant correlation was found between KPS, ECOG status evaluation, QLQ-C30, and QLQ-LC13 in our study. In the study conducted by Langendıjk et al., 71 patients under NSCLC palliative RT were examined. A complete response was observed objectively in eight patients; significant improvement in dyspnea and fatigue was detected in patients with complete response [18]. However, in our study, the complete answer was dyspnea, fatigue, hemoptysis, and pain symptom palliations. For evaluation of symptom palliation, including symptom scales such as dysphagia, pain, and dyspnea, the efficacy of QLQ LC13 was proven. Dysphagia is expected as a side effect in patients undergoing RT, and it is directly useful with QoL. When a randomized study and the study of Langendıjk were examined, symptom palliation rates were the same; 80% of palliation in chest pain and 50% palliation in dyspnea were observed. According to these studies, cough palliation was fewer; hemoptysis, chest pain, and dyspnea palliations were observed at a higher rate in our study.

In our study, dysphagia scoring was found high in the QLQ-LC13 scoring at the end of RT; it also decreased in the first and second-month follow-ups, and full symptom palliation was achieved spontaneously in the third-month follow-ups. The administration of corticosteroid and bronchodilator treatments to patients at appropriate doses with RT might have played a critical role in the higher rate of dyspnea palliation in our study compared to the literature. Although there is no study in the literature showing that supportive therapy with RT increases QoL, it was shown that supportive treatment with chemotherapy boosts QoL [19-20]. The symptom of fatigue has been considered the most crucial criterion for QoL [21-22]. In our study, ECOG performance status was high. Also, it was observed that there was palliation in the pain symptom in their scores at the beginning and end of RT in patients with lung cancer who underwent concurrent chemoradiotherapy. The pain was reduced in chemotherapy and was found to be in the literature [23]. There was symptom palliation in another randomized trial, similar to our study, especially in cough, hemoptysis, chest pain, and dyspnea in the group that underwent hyperfractionated RT (19). Our study provided more palliation in the dyspnea symptom than the study mentioned above. In the randomized NSCLC dose-escalation trial, RTOG 0617, there was no difference in the third-month follow-ups in 219 patients who were administered 74 Gy and 60 Gy using conformal RT or IMRT [24].

It is known that the dose and radiotherapy areas handle the side effects of RT. The radiotherapy area is associated with QoL [25]. Side effects and QoL depend on the RT area [16]. When the mean values of QoL of males and females are examined in our study, it is seen that the number of female patients is deficient as compared to the number of male patients, and there is a significant increase in their mean scores in the third month as compared to their QoL score at the beginning. However, there is a decline in the third-month scores of male cases as compared to their scores at the beginning of RT. Studies in the literature showed differences between genders and differences in the mean values of QLQ scores, similar to our study [26]. A study evaluated 100 patients with head and neck cancer using EORTC QLQ-C30 and QLQ-H&N35; no significant relationship was found between QoL parameters and genders. In another study, gender was associated with loss of appetite, pain, and financial difficulties, especially in QLQ-C30 [16]. Higher global scores were obtained in stage III patients in the studies, and lower global scores were obtained in patients with stage I and II. They attributed this to the consideration of stage I-II cases as medically inoperable because of co-morbid diseases [27]. Although it is known that the triple treatment modalities administered in lung cancer cases are toxic, the study of Schumacher et al. demonstrated that the triple modality did not lead to a decrease in QoL in stages IIb and III NSCLC [28]. Similarly, in our study, symptom palliation and the global health score of stage III patients were higher than those of stage I and II. In the relationship between QoL and prognostic factors in lung cancer, QoL was a predictive and prognostic factor with weight loss in the literature [29].

## Conclusions

During the study after treatment with radiotherapy for lung cancer, the QoL level was maintained: There was a decline of the global health status (p < 0.000). The physical functioning score and the role function score increased, which was also significant (p < 0.002). Tumor reduction plays a role in the palliation of some respiratory symptoms, except hemoptysis.
